# Wireless In-Ear Communication for Total Joint Arthroplasty: A Simulated Operating Room Evaluation

**DOI:** 10.1016/j.artd.2024.101481

**Published:** 2024-08-12

**Authors:** Blake T. Dunson, Alexus M. Cooper, Bryce W. Polascik, Taylor R. Wood, Maxwell K. Langfitt, Johannes F. Plate, Samuel Rosas

**Affiliations:** a Department of Orthopaedic Surgery, Atrium Health Wake Forest Baptist, Winston-Salem, NC, USA; b Wake Forest University School of Medicine, Winston-Salem, NC, USA; c Department of Orthopaedic Surgery, University of Pittsburgh Medical Center, Pittsburgh, PA, USA

**Keywords:** Arthroplasty, In-ear communication, Orthopaedic surgery noise, OR communication

## Abstract

Effective communication is vital for patient safety, yet failures are common, often due to outdated methods. This study aimed to assess whether in-ear communication devices improve communication in orthopedic surgery simulations compared to traditional loud voice methods. Fifteen participants underwent simulations using both in-ear wireless devices and standard communication. Results showed significant improvements with in-ear devices in correctly identifying phrases (78.6% vs 44%), effectiveness (7.9/10 vs 4.9/10), and clarity (8/10 vs 4/10), all *P* < .001. Participants also favored in-ear devices in usability assessments. Sound levels recorded were comparable between groups. In conclusion, in-ear communication is safe and effective in orthopedic settings, potentially enhancing efficiency and safety. These devices can mitigate loud noises, benefiting surgeon well-being and patient outcomes.

## Introduction

Communication breakdown plays a significant role in the occurrence of adverse events across many healthcare domains. In its examination of 4000 adverse events, the Joint Commission specifically pinpointed communication breakdown as the most common contributing factor to these incidents [1]. In the operating room (OR), communication failure contributes to 43% of errors [2]. In spite of this, OR communication is an area that has not been deeply studied [3]. Moreover, the importance of rapid, efficient and effective communication within surgical teams is a cornerstone for successful outcomes [4,5]. In the high-stakes environment of the OR, miscommunication or breakdowns in information exchange can lead to a cascade of adverse events. Surgical errors, procedural delays, and compromised patient safety are among the immediate consequences that may arise [6]. In addition to immediate consequences, such breakdowns can have long-term impacts on OR efficiency, cost, and increase tension between surgical teams [[7], [8], [9]].

Orthopaedic surgery, a field with heavy use of saws, hammers, and whole-body sterile suits is a field where communication can be hampered by the noises of the surgery and the processes within it [[10], [11], [12]]. Improving conversational efficiency within teams has the potential to improve OR efficiency, resident education, and most importantly, patient outcomes. Nonetheless, this area of orthopaedics has been less studied and has lacked innovation that could potentially alleviate miscommunication within the teams. Additionally, communication tools that facilitate quick team interaction can also hamper the loud noises in arthroplasty surgery. Numerous studies have indicated that the significant noise levels experienced by orthopaedic surgeons puts them at a higher risk of noise-induced hearing loss [[13], [14], [15]]. A previous study by Kwan et al. demonstrated that orthopaedic surgeons are exposed to noise-induced hearing loss with daily levels above 85 decibels and that arthroplasty surgeons were the ones with the highest dose and highest projected dose among orthopaedic subspecialties. Furthermore, a recent study from Germany demonstrated that surgeons exposed to robotic arthroplasty are exposed to sound levels above the recommended dose from the National Institute for Occupational Safety and Health [16]. These high sound levels were further validated by the Noise Evaluation of Arthroplasty Theaters study which demonstrated that surgeons in the United Kingdom exceeded noise level recommendations from their national regulating agency when performing total hip arthroplasty [17]. Some have even recommended that surgeons be included in a hearing loss prevention program [10,14]. Therefore, the objective of this study was to assess whether the utilization of in-ear communication devices during simulated orthopaedic surgery procedures can enhance communication efficiency, dampen the level of sound exposure with in-ear technology, and determine the practicality of implementing such a system.

## Material and methods

### Demographics

A total of 15 volunteers participated in the study. Most participants were women (9/15, 66%). The mean age of participants was 29 years (standard deviation [SD] 2.9). No participants had a history of hearing problems, and all were either residents or medical students.

### Simulation design

**Figure 1:**
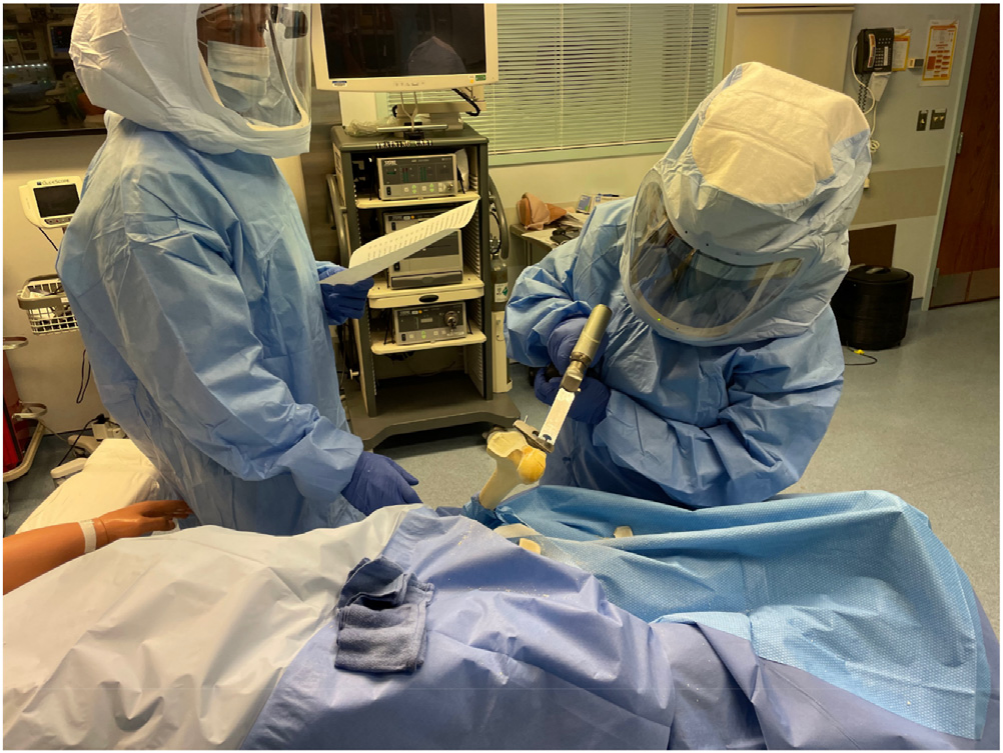
Total joint arthroplasty simulation.

To emulate the OR environment, a simulation of total joint arthroplasty was orchestrated (Fig. 1). This simulation, conducted at the mock OR of the Center for Applied Learning at Atrium Health Wake Forest Baptist Hospital, encompassed the essential elements of a real OR, incorporating saw bones (Sawbones USA, Vashon, WA), requisite instruments, standard procedures, draping, setup, and conditions specific to TJA. Furthermore, the simulation emulated the authentic OR experience, incorporating gowning with sterile suits and the utilization of the anesthesia machine and suction device in simulation mode, reproducing typical OR ambient noise. The level of noise was confirmed prior to the initiation of the simulations based on previous studies [18]. The Center for Applied Learning system served as the cornerstone for the study's simulations, where different phases of the TJA case were examined. Critical procedural stages where loud noises are encountered were targeted for comparison including pin placement into cutting blocks and bone cuts in saw bones using a standard oscillating saw. The use of the oscillating saws was targeted as the loudest portion of the procedure and thus chosen to be compared. Volunteer residents and medical students were recruited to participate in the simulations.

### Center for Applied Learning case simulations

Participants were randomly tested in 2 different case simulations:

1) OR simulation without in-ear communication: Participants used verbal communication to interact with standard OR noise while cutting a bone saw model wearing hooded protective OR suits with the helmet fan engaged (Flyte Personal Protection System, Stryker Corp. Portage, MI).
2) OR simulation with in-ear communication: Participants used in-ear communication devices to interact with standard OR noise while cutting a bone saw model wearing again personal protective suit.

### Data acquisition

The primary outcomes assessed were communication effectiveness and sound level exposure. Communication evaluation was based on the study by Thomas et al. [18] and was determined by the subjects' ability to execute tasks as instructed by the moderator without the need for task repetition. To capture sound data during simulation, in ear devices with decibel recording and wireless communication capabilities were used. A set of current generation Apple AirPods (Apple, Cupertino, CA) were used as the in-ear device. For the purposes of this study, 2 current generation iPhones (Apple, Cupertino, CA) were used. These were connected to the hospital's non-password-protected guest WIFI network and the Discord app (Discord Inc, San Francisco, CA) was downloaded in both phones. The app allows the creation of private communication rooms of users that have a profile within the app and are online. The creation of a connectivity channel allows the users to speak "over the phone" to each other through a secure network. The internet protocol addresses of each user are securely protected online by being routed through a Discord server. Creation of a profile within the app takes less than a minute. The app Decibel: dB sound level meter (Vlad Polyanskiy, iOS App Store) on an Apple iPhone 12 (Apple, Cupertino, CA) was used. Sound recordings were analyzed within the sterile suite and through the in-ear device while in use. Peak values and cumulative noise exposure levels were quantified, analyzed, and compared across case simulations.

The critical portion of the procedure evaluated was the cutting of the distal femur with an oscillating saw (System 7, Stryker, Portage, MI) and 1.27 mm blade (Stryker, Portage, MI). Femur Saw Bones were used (Sawbones USA, Vashon, WA). The distal cutting guide for a total knee arthroplasty was used with smooth pins drilled in place (Depuy Attune, Johnson & Johnson, Warsaw, IN).

**Figure 2:**
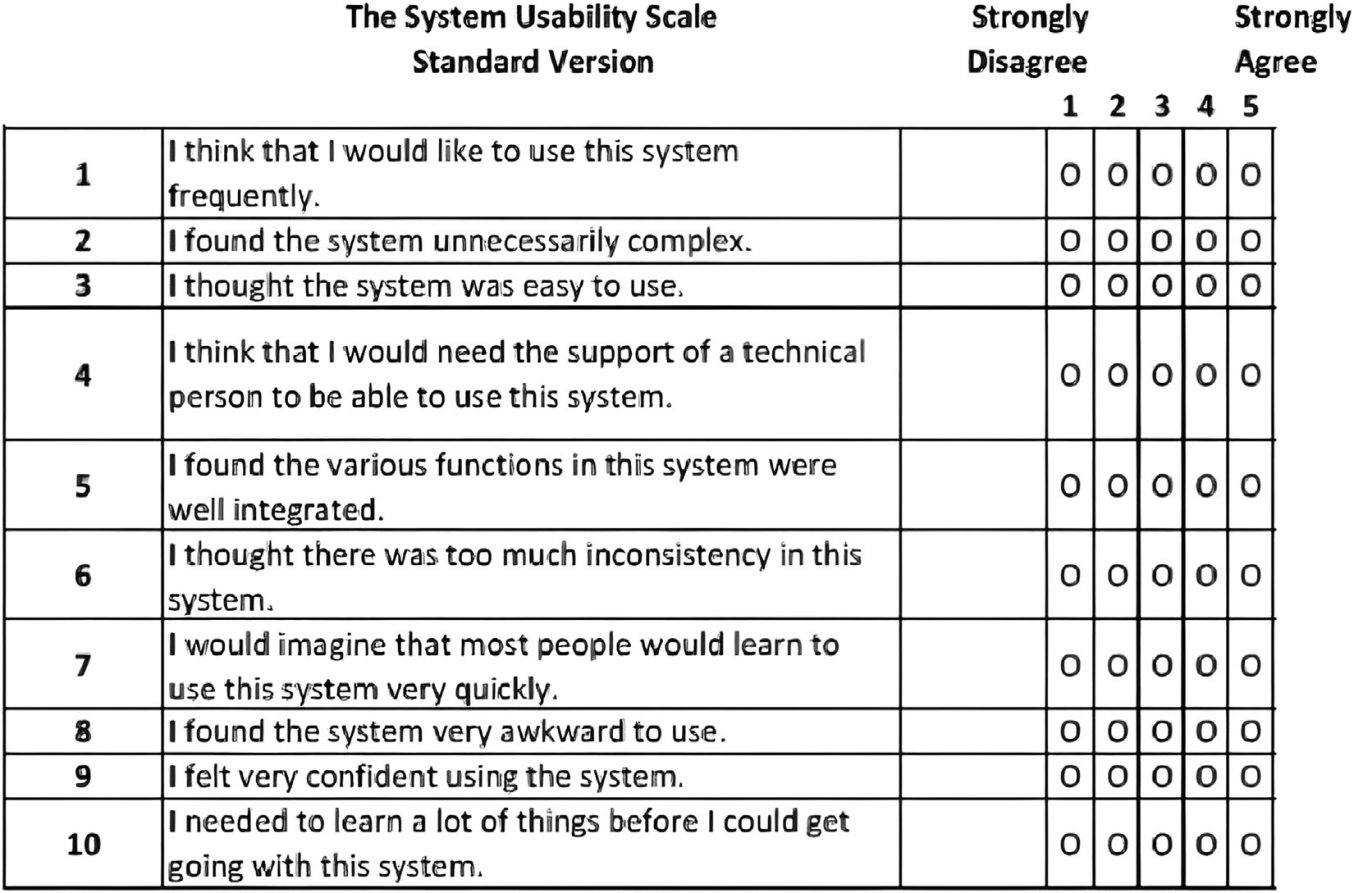
The system usability score questionnaire.

Secondary outcomes included participants' clarity and effectiveness ratings following each simulation. Clarity was defined as the amount of static, interference, or the quality of transmission between the moderator and participant. Effectiveness was defined as whether participants thought the modality was practical to use in the OR. Ratings for clarity and effectiveness were assigned on a visual analog scale ranging from 1 to 10, with 1 denoting the worst and 10 signifying the best [19]. Finally, following completion of a participant’s final case simulation, participants completed a System Usability Scale (SUS) [20] to determine satisfaction with each type of communication. The SUS is a questionnaire consisting of 10 items that evaluate the perceived usability of a system. Participants rated the 10-item SUS questionnaire (Fig. 2) on a scale from 1 to 5, with responses corresponding to the following values: Strongly Disagree (1 point), Disagree (2 points), Neutral (3 points), Agree (4 points), and Strongly Agree (5 points). Additionally, participants were given a questionnaire evaluating opinions regarding ease of use, preferred method, problems encountered, possible barriers in implementation of intraear communication devices, and whether they anticipate a future transition to in-ear devices. Finally, an open question on “thoughts” on the experience was offered to further capture user experience.

### Phrases used

To make the simulations as close to the operating room as possible, phrases that are typically used within a TJA procedure were printed onto sheets that participants were blinded to until the simulation took place. They are presented on Table 1. Phrases no longer than 5 words were used to avoid adding more variables to the study. Enough phrases were made available, so the participants did not have to repeat phrases when performing the second case simulation to prevent recollection bias.

**Table 1:** Phrases used for the communication assessment.

Option number	Phrases
1	1. More suction please 2. Raise your hand 3. New drill needed 4. More traction please 5. Call next case 6. Irrigation please 7. Drop your hand 8. Size 5 fits 9. Open the cement 10. Clean the flutes
2	1. Retract more patella 2. Raise your machine 3. Drop the leg 4. Blood pressure down 5. Need more sawing 6. Mallet please 7. Move the pin 8. Add more force 9. Leave the pin 10. Block more distal
3	1. Get the bovie 2. Use the knife 3. Cut more femur 4. Take more tibia 5. Pin remover next 6. Need pain cocktail 7. Get injection ready 8. Take the hammer 9. Move the drill 10. Antibiotics given?
4	1. Need more dissection 2. Increase flexion gap 3. Decrease flexion space 4. Patient doesn’t smoke 5. Patient is very large 6. Surgery as outpatient 7. Will need PT 8. give tranexamic acid 9. Need bigger poly 10. Need smaller poly

### Data analyses

Descriptive analysis was performed followed by qualitative. Initial analysis of data parametricity was evaluated with the Kolmogorov-Smirnov test. Based on normality testing the variables were compared later using *t-tests*, while *chi-squared* tests were used for the comparison of qualitative variables, with a significance level set at *P* < .05. The data collected from the simulations were compared to determine the effectiveness of in-ear communication and sound level exposure in the OR. Qualitative recording of participants' comments after the study was also saved and presented herein.

### Source of funding

No outside funding was provided for this study. All Sawbones used for the study were provided by the Atrium Health Wake Forest Baptist Orthopaedic Surgery Research Department. Institutional review board approval was obtained for this study and each participant signed an informed consent form. Michael McCann helped provide the instruments needed for the simulation and Dr Johnson and his team provided assistance with the set-up of the Center of Experiential and Applied Learning and we greatly thank them.

## Results

### Communication effectiveness

There were 5 phrases spoken to each participant in each simulation. There was a significant difference in percent of correctly identified.

Both effectiveness and clarity were ranked significantly higher for the in-ear communication device simulations. The mean effectiveness for the in-ear communication simulation was 7.9/10 (SD 1.94) compared to 4.9/10 (SD 2.12) for the traditional communication simulation (*P* < .05). The mean clarity for the in-ear communication simulation was 8.0/10 (SD 1.85) compared to 4.0/10 (SD 2.29) for the traditional communication simulation (*P* < .05).

Regarding the SUS questionnaire, significant differences were observed in the means for 4/10 of the SUS questions (Table 2). Ten participants filled out the additional survey at the end of their final simulation. Of these, 9 preferred and anticipated a future transition to an in-ear communication system. The most positive and negative open response comments are shown in Table 3.

**Table 2:** Comparison of means and standard deviations for the system usability score questionnaire.

Headphone		No headphone		*P* value
Mean	SD	Mean	SD	
4.53	0.83	3.00	1.18	.000
1.79	0.89	2.36	1.45	.220
4.27	0.96	3.36	1.22	.033
1.8	1.01	2.00	1.52	.678
3.87	1.13	2.86	1.17	.025
2.2	1.08	3.43	1.40	.013
4.47	1.06	4.14	1.03	.411
1.73	0.88	2.64	1.34	.038
4.13	0.92	3.43	1.22	.089
1.6	0.63	1.93	1.44	.428

**Table 3:** Free-text responses regarding the most-positive and most-negative aspects of the in-ear communication simulation.

Most-positive aspects	Most-negative aspects
“Reliable”	“Intolerance of headphones”
“Noise Canceling”	“Delayed voice transmission”
“Great idea”	“Headphones dying”
“Could see the OR having a complete audio system for the whole team”	“AirPods could fall off in hood and would be hard to retrieve”
“Effective”	“Connectivity trouble and Cutting out”

OR, operating room.

### Sound level exposure

Mean level of sound recorded was 86 dB in the in-ear device and 87.6 dB in the control group which demonstrated no difference (*P* = .680).The peak level of sound was recorded at 99 dB across all the simulations.

## Discussion

Despite effective communication being critical for outcomes in the OR [1,2,4], there has been a notable lack of innovation in this area. Specifically in orthopaedic, power tools that emit loud noises are utilized and personal protective suits with fans are common that can inhibit effective team communication [[10], [11], [12],^21^]. Wireless in-ear communication systems have been proposed as a potential solution to this problem. In this study, we present the first randomized simulation study evaluating an in-ear communication system in orthopaedic surgery.

With a sample of 15 participants, the results indicate that in-ear wireless communication under OR conditions appears to be safe and effective. Participants in the in-ear simulation demonstrated a significantly higher number of accurately identified phrases, along with improved effectiveness and clarity. Additionally, feedback from the SUS questionnaire and survey indicated that participants found the system to be effective and easy to implement.

These findings do not come as a surprise given the demonstrated benefit of in-ear communication systems in aviation [22], professional sports teams, and healthcare teams [3,23]. In regard to OR communication, previous studies have shown benefit with wireless communication systems yet none in the setting of TJA [8,18,24,25]. In a prospective blinded study, Thomas et al. [18] conducted a comparison between communication efficiency using the standard Da Vinci Si speaker system and a wireless, hands-free audio system. The evaluation involved subjects hearing 960 surgical phrases and transcribing them onto a data sheet. The findings indicated that the wireless, hands-free system led to increased communication accuracy, clarity, and effectiveness. Ortega et al. conducted an evaluation of paging communication using an in-ear communication device. In a randomized approach, the orthopaedic surgeon was paged during procedures both with and without this device. The study revealed noteworthy improvements in meantime intervals for response time, correct patient identification, and total communication time. Additionally, there were enhanced satisfaction ratings from floor nurses and a reduction in intraoperative case interruptions [8].

Furthermore, wireless communication systems bypass personal protective equipment, a known barrier to effective communication, resulting in improved speech discrimination and decreasing listening effort [24,25]. These systems also provide valuable protection from the high noise levels in the OR, a significant concern in the field of orthopaedics where noise levels can exceed 100 decibels (dBA) for more than 40% of the time during procedures [26]. Additionally, equipment-related noises, such as the use of electric or air-powered surgical instruments and hammers, can reach up to 120 dB [26]. In our study, the mean sound level recorded in the in-ear device was 86 dB and peaked at 99 db.

This study has many potential implications. First, an in-ear communication system can improve efficiency in the OR by decreasing operative times, intraoperative interruptions, surgical delays, and resource utilization [[7], [8], [9]]. Most importantly, the implementation of such a system has the potential to enhance patient outcomes by reducing errors, preventing near misses, and ultimately leading to improved patient safety.

This study is the first of its kind in the field of TJA to demonstrate the effectiveness of in-ear OR communication; however, it is not without limitations. First, the in-ear simulation accurately identified 78.6% of phrases, indicating there is still considerable room for improvement in the system. The in-ear communication system relied on internet connectivity in the operating room, and some participants reported issues such as connectivity problems and interruptions in the in-ear system, potentially accounting for the approximately one-fifth of incorrect responses. Addressing these concerns could be a focus for future in-ear communication systems designed for the operating room. Second, our experimental model used a simulated OR, which may not completely replicate every noise and distraction encountered in a real OR. Third, we focused on testing a single type of orthopaedic procedure in our simulation. Furthermore, participants had a relatively low mean age, potentially underestimating the influence of presbycusis with the increasing age of the surgeon population. While this study has shown that in-ear communication can be more effective than traditional methods, it is crucial to undertake future research involving various procedures and surgical specialties to fully validate this finding. Another limitation of our study was that the study subjects used were medical students and residents of all training years which translates into limited experience performing arthroplasty surgery. As such some of the phrases used in the study may not been familiar to the study subjects. We aim for future studies to include Attending Surgeons and OR staff including circulators and scrub technicians so our findings can be further explored. Finally, another limitation to our study is that our simulations were made to match as closely as we could most joint arthroplasty surgeries done in the United States. Most surgeons use protective hoods that would hold the headphones outside of the surgical field if they were to fall but we did not perform simulations of the cases where protective hoods are not used. Despite these limitations, this study emphasizes the gap of innovation in OR communication and the increased concern for noise induced hearing loss among surgeons who are projected to perform even higher numbers of arthroplasty cases and growing trend in robotic use. Given the demonstrated negative impact on patient outcomes associated with inadequate communication, it is paramount for enhanced innovation in this realm, particularly within the field of orthopaedics.

## Conclusions

Successful communication in the operating room is critical for favorable outcomes and stands as a primary factor influencing patient outcomes. This study highlights a significant enhancement in communication through the implementation of an in-ear communication system, suggesting its potential to reduce errors, instances of miscommunication, and OR efficiency resulting in improved patient safety.

## Conflicts of interest

The authors declare there are no conflicts of interest.

For full disclosure statements refer to https://doi.org/10.1016/j.artd.2024.101481.

## CRediT authorship contribution statement

**Blake Dunson:** Writing – review & editing, Writing – original draft, Data curation. **Alexus M. Cooper:** Writing – review & editing, Data curation. **Bryce W. Polascik:** Writing – review & editing, Investigation, Data curation. **Taylor Ranae Wood:** Writing – review & editing, Project administration, Investigation, Data curation. **Maxwell K. Langfitt:** Writing – review & editing, Supervision, Resources, Funding acquisition. **Johannes F. Plate:** Writing – review & editing, Supervision, Conceptualization. **Samuel Rosas:** Writing – original draft, Project administration, Methodology, Investigation, Formal analysis, Conceptualization.

## Appendix A Supplementary data

Conflict of Interest Statement for Cooper

Conflict of Interest Statement for Dunson

Conflict of Interest Statement for Polascik

Conflict of Interest Statement for Plate

Conflict of Interest Statement for Langfitt

Conflict of Interest Statement for Rosas

Conflict of Interest Statement for Wood
